# Brain Alterations Linked to the MPTP Mouse Model of Parkinson's Disease Uncovered by Diffusion Kurtosis Imaging and Magnetic Resonance Spectroscopy

**DOI:** 10.1002/cns.70846

**Published:** 2026-04-20

**Authors:** Ajay Modi, Sheetal Maria, Jana Ruda‐Kucerova, Eva Drazanova, Iveta Harastova‐Pavlova, Alzbeta Sejnoha Minsterova, Kristina Kovacovicova, Daniel Havas, Irena Rektorova, Tiago F. Outeiro, Amit Khairnar

**Affiliations:** ^1^ Center for Translational Medicine, International Clinical Research Centre St. Anne's University Hospital Brno Czech Republic; ^2^ International Clinical Research Centre, Faculty of Medicine Masaryk University Brno Czech Republic; ^3^ Department of Pharmacology, Faculty of Medicine Masaryk University Brno Czech Republic; ^4^ Institute of Scientific Instruments of the Czech Academy of Sciences Brno Czech Republic; ^5^ Applied Neuroscience Research Group, CEITEC ‐ Central European Institute of Technology Masaryk University Brno Czech Republic; ^6^ Psychogenics Inc Paramus New Jersey USA; ^7^ University Medical Center Göttingen Department of Experimental Neurodegeneration, Center for Biostructural Imaging of Neurodegeneration University Medical Center Göttingen Göttingen Germany; ^8^ Translational and Clinical Research Institute, Faculty of Medical Sciences Newcastle University Newcastle Upon Tyne UK; ^9^ Faculdade de Medicina e Ciências Biomédicas, Algarve Biomedical Center Research Institute Universidade do Algarve Faro Portugal; ^10^ Deutsches Zentrum für Neurodegenerative Erkrankungen (DZNE) Göttingen Germany; ^11^ Department of Physiology, Faculty of Medicine Masaryk University Brno Czech Republic

**Keywords:** diffusion kurtosis imaging (DKI), microstructural changes, MPTP animal model, neurodegeneration, Parkinson's disease, proton magnetic resonance spectroscopy (^1^H‐MRS)

## Abstract

**Aims:**

This study employed diffusion kurtosis imaging (DKI) and proton magnetic resonance spectroscopy (^1^H‐MRS) on an MPTP‐induced mouse model of Parkinson's disease (PD) to examine microstructural changes linked to neuroinflammation and neurodegeneration.

**Methods:**

MPTP (20 mg/kg, i.p.) was given for 4 days, and behavioral assessment, MRI imaging, and immunohistochemistry were performed at 24 h and 72 h after last MPTP treatment.

**Results:**

At 24 h, DKI showed higher diffusivity metrics in the hippocampus and thalamus, while ^1^H‐MRS identified reduced Glu/tCr and Glx/tCr ratios in the striatum of MPTP‐treated mice compared to saline‐treated mice. Behavioral tests at 72 h revealed motor impairment and DKI showed increased diffusivity in the somatosensory cortex, thalamus, and striatum in MPTP‐treated mice. Notably, at 72 h, the hippocampus showed partial recovery in diffusivity, suggesting adaptive changes or partial restoration. Higher diffusivity was observed in the cortex, striatum, and thalamus in MPTP‐treated mice. Furthermore, ^1^H‐MRS detected a higher Tau/tCr in the striatum, while in the hippocampus, lower Gln/tCr and NAA/tCr and higher Cho/NAA were observed at 72 h in MPTP‐treated mice, indicating persistent neuronal death and membrane deterioration. Immunofluorescence staining at 72 h confirmed these findings, showing a decrease in NeuN+ neurons and an increase in GFAP+ glial cells in the striatum and hippocampus, indicating neurodegeneration and gliosis. Additionally, MPTP caused a loss of dopaminergic neurons in the substantia nigra and striatum, which likely explains the higher diffusivity shown by DKI.

**Conclusion:**

These findings demonstrate DKI and ^1^H‐MRS are sensitive, non‐invasive modalities for detecting and monitoring neurodegenerative microstructural and neurochemical changes, enhancing the understanding of PD‐related pathology and progression.

## Graphical abstract

In MPTP‐treated mice, DKI and ^1^H‐MRS revealed increased hippocampal and thalamic diffusivity with reduced striatal Glu/tCr and Glx/tCr at 24 h. By 72 h, cortical, striatal, and thalamic diffusivity rose, with partial hippocampal recovery. Elevated Tau/tCr and hippocampal metabolite alterations paralleled neuronal loss and gliosis, supporting DKI/^1^H‐MRS as sensitive biomarkers of progression.

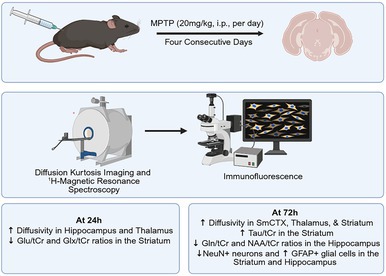

**Abbreviations**
AD: axial diffusivity
AK: axial kurtosis
DA: Dopamine
DKI: Diffusion Kurtosis Imaging
FA: Fractional Anisotropy
GFAP: Glial Fibrilary Acidic Protein
Gln: Glutamine
Glu: Glutamate
Glx: Glutamine‐Glutamate
HIPP: hippocampus
IBA1: Ionized‐calcium Binding Adapter molecule 1
MD: Mean Diffusivity
MK: Mean Kurtosis
MPTP: 1‐methyl‐4‐phenyl‐1,2,3,6‐tetrahydropyridine
MRI: Magnetic Resonance Imaging
MRS: Magnetic Resonance Spectroscopy
NAA: N‐Acetyl Aspartate
Cho/NAA: Choline/N‐Acetyl Aspartate
NeuN: Neuronal Nuclear Protein
OFT: Open Field Test
6‐OHA: 6‐hydroxy dopamine
PD: Parkinson's Disease
RD: Radial Diffusivity
RK: Radial Kurtosis
ROI: Region of Interest
SD: Standard Deviation
SEM: Standard Error Mean
SN: Substantia Nigra
STR: Striatum
Tau: Taurine
TBSS: Tract Basis Spatial Statistic
THAL: Thalamus

## 1 Introduction

Parkinson's disease (PD) is the second most prevalent neurodegenerative disorder after Alzheimer's disease [1]. The pathological features of PD include the loss of dopaminergic neurons in the pars compacta of the substantia nigra (SN) and the accumulation of α‐synuclein (α‐syn) in intraneuronal inclusions known as Lewy bodies and in Lewy neurites [2, 3]. At present, the diagnosis of PD and related disorders is based on clinical assessment, but there is hope that with the development of novel, less invasive biomarkers and imaging approaches we might be able to improve the diagnostic accuracy [4] and anticipate disease severity, monitor disease progression, and evaluate the efficacy of therapeutic interventions. In this context, the development of sensitive imaging biomarkers for early‐stage PD detection is crucial.

For research purposes, hyperphosphorylated α‐synuclein can now be detected in vivo in the CSF and skin biopsy using seed amplification assays; however, so far, this method provides only a yes or no answer and does not allow monitoring disease progression in time [5, 6]. Recently, based on the complexity and heterogeneity of PD, 3 3‐component system, a new biological classification system has been proposed called SynNeurGe. It is suggested first to test the presence and absence of pathological α‐syn in tissues/CSF (S), neurodegeneration defined by neuroimaging approaches (N), and detection of pathogenic gene variants (G). The current neuroimaging approaches are FP‐CIT SPECT for whole dopaminergic imaging and MIBG heart scintigraphy. While for MRI, free water imaging, neuromelanin sensitive MRI, and iron sensitive MRI, like quantitative susceptibility mapping and susceptibility weighted imaging, which image dorsal nigral hyperintensity. These SPECT, PET, and MR imaging techniques need to be harmonized to prove it as a clinical biomarker [6]. Potential techniques for evaluation of subtle microstructural changes in the brain at various time points include functional MRI, proton magnetic resonance spectroscopy (1H‐MRS), and diffusion kurtosis imaging (DKI). DKI is an extension of diffusion tensor imaging (DTI), comprehensively characterizing water diffusion in tissues [7, 8]. ^1^H‐MRS provides valuable insights into the brain's neurochemical environment by detecting and quantifying various metabolites. It measures explicitly proton‐containing compounds and has become a pivotal tool in neuroscience research [9].

Several studies have demonstrated the utility of DKI in detecting microstructural changes in the brains of PD patients and animal models. For instance, DKI has revealed reduced kurtosis metrics in the SN and other basal ganglia regions in PD patients, correlating with disease severity and duration. This reduction in kurtosis is thought to reflect the loss of cellular complexity and density due to neurodegeneration [10]. While some clinical DKI studies have found increased diffusion kurtosis metrics in cognitively normal PD patients reflecting increased hindrance to water diffusion due to α‐syn induced microstructural changes [11]. Additionally, changes in kurtosis have been associated with alterations in axonal integrity and myelin breakdown, further highlighting the potential of DKI as a biomarker for PD [12]. In animal models, DKI was able to detect α‐syn induced microstructural changes in a transgenic mouse model of PD (TNWT‐61) [13] as well as neurodegeneration in neurotoxin (methamphetamine and rotenone) based animal models of PD [14, 15].

Several studies have employed ^1^H‐MRS to investigate metabolic changes in PD patients and animal models. In various clinical studies, ^1^H‐MRS was used to examine PD patients' metabolic alterations in the SN and striatum (STR). The researchers found significantly reduced NAA (N‐Acetyl Aspartate)/Cr and Cho (Choline)/NAA ratios in these regions, suggesting neuronal loss and dysfunction. This study highlighted the potential of ^1^H‐MRS to detect neurochemical changes that precede overt structural atrophy, thereby providing early biomarkers for PD [16, 17, 18]. In addition, ^1^H‐MRS has elucidated the biochemical changes in animal models of PD associated with neurodegeneration. For instance, Boska et al. (2013) utilized ^1^H‐MRS to assess metabolic changes in the brains of 1‐methyl‐4‐phenyl‐1,2,3,6‐tetrahydropyridine (MPTP)‐treated mice, a widely used animal model of PD. The study revealed decreased levels of NAA in the SN, reflecting neuronal loss and impaired neurotransmission. These findings were corroborated by histological analysis, which showed dopaminergic neuronal loss, thereby validating the ^1^H‐MRS results [19].

The classical MPTP mouse model is more popular for PD research as it shows progressive neurodegenerative changes. It has been reported with the chronic MPTP mouse model that after 1st administration of MPTP, the mice showed neuroinflammatory changes; however, the neurodegeneration begins immediately after MPTP administration, but the extent of damage becomes significant at different time points depending on the regimen. In this study, it was found that neurodegenerative changes became significant after the 7th MPTP dose and further intensified by the 10th dose, illustrating the progressive nature of MPTP model [20]. Moreover, the subacute systemic MPTP regimen (20 mg/kg, i.p., once daily for 4 days) is widely employed. As it produces a robust and reproducible dopaminergic lesion in C57BL/6 mice while minimizing mortality and excessive systemic burden. Several studies have demonstrated that this dosing schedule leads to rapid biochemical and microstructural alterations within hours, followed by progressive dopaminergic terminal loss over the subsequent days [21, 22].

It reliably triggers early mitochondrial dysfunction, oxidative stress, gliosis, and striatal dopamine depletion without the systemic toxicity associated with acute or prolonged regimens. Although injury begins after the first dose, cumulative exposures intensify degeneration, particularly after the third to fourth injection [23, 24]. These suggest that this regimen provides an optimal balance between reproducible early neuroinflammatory and neurodegenerative changes.

Therefore, in the present study, we aimed to detect the neuroinflammatory changes at the 24‐h time point and neurodegenerative changes at 72 h after MPTP administration using MRI in C57/BL6 mice. To date, no study has used the integration of DKI and ^1^H‐MRS to identify PD‐like changes from a microstructural and biochemical perspective. Together, these techniques can elucidate the complex interplay between microstructural damage and metabolic dysfunction, enhancing our understanding of disease progression. Integrating these imaging techniques with behavioral assessments and histological validation provides a comprehensive approach to reveal the progression of PD‐like pathology.

## 2 Material and Methods

### 2.1 Animals

Male C57BL/6 mice (12 weeks old) were obtained from Charles River, Germany. They were housed in the Masaryk University experimental animal facility under controlled conditions with a room temperature of 23°C ± 2°C, a 12‐h dark–light cycle (6 AM to 6 PM), and ad libitum access to food and water. All procedures were conducted by EU Directive No. 2010/63/EU and received approval from the Animal Care Committee of the Faculty of Medicine, Masaryk University, Czech Republic, the Animal Care Committee of Czech Academy of Sciences, Czech Republic, and Czech Governmental Animal Care Committee, in compliance with Czech Animal Protection Act No. 246/1992. Animal studies are reported in compliance with the ARRIVE guidelines [25].

### 2.2 Drugs and Reagents

MPTP (1‐methyl‐4‐phenyl‐1,2,3,6‐tetrahydropyridine) hydrochloride was acquired from Selleck Chemicals (Germany) and prepared in 0.9% saline solution for intraperitoneal administration at a dose of 20 mg/kg in 1 mL.

### 2.3 Antibodies

The polyclonal antibody rabbit anti‐tyrosine hydroxylase (TH) (Sigma‐Aldrich #ab152), polyclonal antibody chicken Neuronal Nuclear Protein (NeuN) (Sigma‐Aldrich # ABN91), polyclonal antibody rabbit anti‐Glia Fibrilary Acidic Protein (GFAP) antibody (Abcam, #AB7260) and Recombinant Monoclonal antibody rabbit anti‐Ionized Calcium Binding Adaptor Molecule 1 (IBA1) (Abcam #Ab178846) antibodies were obtained from Sigma‐Aldrich and Abcam respectively, while the secondary antibodies, Alexa Fluor 555 donkey anti‐rabbit IgG (Invitrogen, #A31572), and Alexa Fluor 647 goat anti‐chicken IgY (Invitrogen, #A21449) were acquired from Invitrogen, Thermo Fischer. DAPI (Merck, #10236276001), Protein block (Abcam, #Ab647226), and Antibody diluent (Abcam, #Ab64211) were also purchased from Abcam. All reagents utilized in this investigation were of analytical and molecular grade.

### 2.4 Experimental Design

The experimental animals (*N* = 30) were randomly allocated into the MPTP‐treated (*N* = 15) and saline‐treated (vehicle) control (*N* = 15) groups. MPTP or vehicle was administered daily to the mice for four consecutive days. There were two temporal points for assessment: 24 h to detect neuroinflammatory changes and 72 h to detect neurodegeneration.

24‐h time‐point (*N* = 12, for each group *N* = 6): 24 h following the last MPTP treatment administration, the mice underwent DKI and ^1^H‐MRS scanning, after which they were transcardially perfused.

**FIGURE 1:**
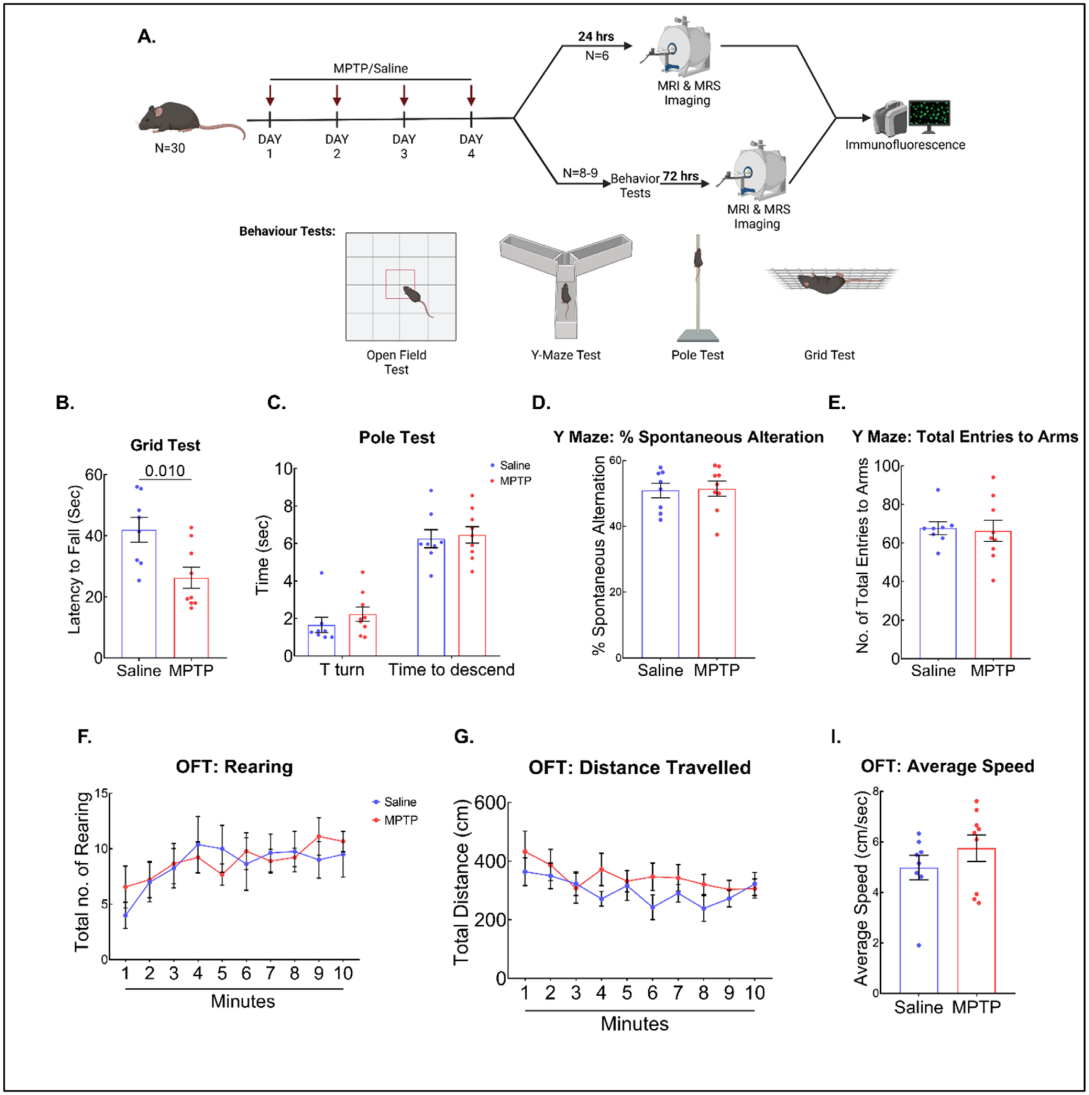
Effect of MPTP on motor and cognitive function. (A) Schematic representation of experimental timeline of the study; (B) Grid Test: MPTP induces a significant reduction in latency to fall of mice compared to the saline. (C) Pole Test: MPTP treatment did not alter the time of T turn and descend. (D‐E) Y Maze: MPTP treatment did not affect the total entries to arms and % of spontaneous alternations. (F‐I) OFT: MPTP administration did not affect rearing, distance traveled, and average speed of mice in OFT. Data were reported as mean ± SEM (*n* = 7–9).

72‐h time‐point (*N* = 18, for each group *N* = 9): 72 h after the last MPTP treatment, the mice were subjected to behavioral tests for motor and cognitive impairments. Subsequently, these mice were subjected to DKI and ^1^H‐MRS imaging, after which they were transcardially perfused (Figure 1A).

### 2.5 Behavioral Assessment

The Grid Test assessed motor coordination and forelimb function in the mice. This test was found to be highly sensitive in several mouse models of PD [13, 14, 15, 26]. The grid test apparatus comprises a horizontal grid (12 × 12 cm) positioned 20 cm above a table. Individual mice were placed on the grid, which was inverted, and the latency to fall was recorded with a cut‐off duration of 60 s.

The pole test was conducted to assess motor coordination in mice [15, 27, 28]. The apparatus consisted of a 48 cm wooden rod with a 1 cm diameter positioned vertically, wrapped with a sterile knitted bandage. Each mouse was placed head‐upward near the top of the pole. Mice had a training session, and the test consisted of three trials, with an inter‐trial interval of at least 30 min between trials to prevent fatigue. The test was video‐recorded and analyzed by a blinded experimenter. The time to turn head‐downward (T‐turn) and descend to the pole's base was recorded.

The locomotor activity of individual mice was evaluated using the Actitrack system (Panlab, Spain) [14, 26]. Each Plexiglas arena (45 × 45 × 30 cm) was enclosed by two frames fitted with photocells positioned 2 cm and 7 cm above the cage floor. The mice were placed at the center of the arena, and their spontaneous behavior was monitored for 10 min. During this period, horizontal locomotor activity was quantified by the trajectory derived from beam interruptions in the horizontal sensors, and vertical activity was measured by counting the rearing episodes that interrupted the beams of the upper frame.

The Y Maze Test was conducted to evaluate the spatial memory and cognitive function [26, 29]. The apparatus consisted of three identical arms (20 cm long, 5 cm wide, and 15 cm high) arranged at 120° angles. Mice were placed in the center of the arms and allowed to explore the maze freely for 15 min. Each time a mouse entered an arm with all four paws, an entry was recorded. The sequence of arm entries was documented to assess spontaneous alternation behavior, calculated as the percentage of triads (sets of three consecutive entries into different arms) over the total number of possible alternations.

### 2.6 Diffusion‐Weighted MR Data Acquisition

Diffusion‐kurtosis imaging (DKI) and proton magnetic resonance spectroscopy ( ^1^H‐MRS) experiments were performed on a 9.4 T Bruker NMR small animal system, Bruker BioSpec 94/30 USR, equipped with a gradient system delivering up to 660 mT/m. Volume transmitter coil RF RES 400 1H 112/086 QSN and 4‐channel receiver array mouse head coil RF ARR 400 1H M.BR.LIN were used to obtain the signal.

The localizer experiment was used to acquire the pilot images, then the T2‐TurboRARE experiment was performed in all three orthogonal orientations (axial, sagittal, coronal) as a reference for DKI and MRS settings. Parameters in the T2‐TurboRARE were set: 20 × 20 mm field of view (FOV), 256 × 256 matrix size, 0.5 mm slice thickness, number of slices: 21 in axial, 21 in sagittal, and 15 in coronal orientation, two averages, RARE factor of 8, and the repetition time (TR) was 2500 ms. The experiment in each orientation lasted ~3 min.

For the DKI acquisition, diffusion‐weighted images were acquired with a spin‐echo planar imaging sequence (DTI‐EPI30dir) with two segments. 21 axial slices with FOV = 20 × 20 mm^2^, 128 × 128 matrix size, and 0.5 mm thickness were acquired and were set the same as T2‐TurboRARE reference, covering the brain from olfactory bulbs to the cerebellum. The DKI protocol included the acquisition of six b‐values (b = 0, 500, 1000, 1500, 2000, and 2500 s/mm2) along with 30 non‐collinear directions, δ = 4 ms, Δ = 11 ms, with seven averages used for b = 0 acquisition. TR = 3000 ms, and echo time (TE) was set to 27.37 ms. The generalized autocalibrating partially parallel acquisitions (GRAPPA) with an acceleration factor of 2 was used to improve image quality by reducing sensitivity to motion and inhomogeneity of magnetic susceptibility. The length of the DKI experiment was ~15 min.

The ^1^H‐MRS experiment was performed in two brain regions: right hippocampus (RHIPP), left hippocampus (LHIPP), right striatum (RSTR), and left striatum (LSTR). These two regions are selected as there are reports suggesting the MPTP mouse model of PD not only induces classical dopaminergic neurodegeneration in SN and striatum but also induces hippocampal dopamine deprivation [30]. Also, these regions are technically suitable for 1H‐MRS spectroscopy due to their homogeneity and proximity to the receiver array coil. The measured volumes (voxels) were carefully placed in particular regions based on the T2‐TurboRARE images. The size of the voxel was (2.3 × 0.8 × 1) mm^3^ in the hippocampus and (2.2 × 0.9 × 1) mm^3^ in the striatum. The pulse sequence point resolved spectroscopy (PRESS) for the single voxel MRS was used with the following parameters: spectral width 4401.41 Hz, 4096 points, TE = 16.5 ms (TE1 = 8.8 ms, TE2 = 7.7 ms), TR = 2500 ms, 256 averages, 90° Calculated 0.77‐ms‐long excitation pulse, and two 180° Calculated 1.4‐ms‐long refocusing pulses. Outer volume suppression pulses and VAPOR water suppression pulses were applied. First‐ and second‐order shims were applied based on the previously measured B0 map. The quality of the spectra was checked right after each PRESS sequence from the size of the linewidth of the water peak at 50% height [31] and visually during the MRS data post‐processing.

### 2.7 Data Analysis

The acquisition matrix of DKI images was 128 × 128. MRI data were converted to NIfTI from the Dicom format with a dicm2nii MATLAB function. Diffusion data were corrected for eddy currents and motion artifacts to the first non‐diffusion‐weighted image [32]. The following parametric maps were calculated in ExploreDTI v4.8.4. Software [33]: MK (mean kurtosis), AK (axial kurtosis), RK (radial kurtosis), MD (mean diffusivity), AD (axial diffusivity), RD (radial diffusivity), and FA (fractional anisotropy), using the robust estimation of tensors by outlier rejection (RESTORE) fitting method.

#### 2.7.1 Region of Interest (ROI) Analysis

DKI variables were taken into account within the following regions selected based on our previous DKI studies with Tg mice overexpressing α‐syn and rotenone mouse model: two slices from the SN, five slices from the somatosensory cortex, five slices from the STR, four slices from the Hippocampus (HIPP), and two slices from the thalamus (THAL) (Figure S1) [13, 14, 26]. In brief, ROI selection for different brain areas in the b = 0 images was manually drawn using FA maps in ImageJ software, complying with the mouse brain atlas [34].

#### 2.7.2 Tract‐Based Spatial Statistics (TBSS)

White matter analysis was performed using the tract‐based spatial statistics (TBSS) algorithm [35] in the FMRIB software library. Automatic brain extraction was carried out during the processing in ExploreDTI. Brain‐extracted maps were checked one by one visually, and the extraction was corrected manually. TBSS was implemented and modified according to the protocol for rodent brains [36]. Then, the TBSS was used with the following steps: (a) co‐registration of all individual FA maps and identification of the best registration target with the free‐search method; (b) application of the best registration target as a template for final transformations; (c) calculation of the mean FA map and creation of the mean FA skeleton at the threshold of 0.2 that represents the core of all tracts; (d) projection of each mouse's FA data to this skeleton; (e) repetition of the previous steps for all DKI maps. A two‐sample unpaired *t*‐test design was set in a general linear model. A randomize tool for permutation‐based non‐parametric testing with 10,000 permutations and Threshold‐Free Cluster Enhancement was used for multiple comparison correction, and *p* < 0.05 was deemed significant [37]. The results of the TBSS analysis were identified according to the mouse brain atlas [34].

### 2.8 ^1^H‐Magnetic Resonance Spectroscopy

A 9.4 T Bruker NMR small animal system (Bruker BioSpec 94/30 USR) was used for the NMR investigations. It comprises a 4‐channel receiver array mouse head coil (RF ARR 400 1H R.BR. LIN) and a volume transmitter coil (RF RES 400 1H 112/086 QSN). Four voxels—the right STR, the left STR, RdHIPP, and LdHIPP—were analyzed using the ^1^H‐MRS approach. Hippocampal and striatal voxels, respectively, were positioned at −3.08 mm and 1.2 mm from bregma, respectively [34]. White matter signal detection was prevented by carefully positioning the voxels inside the designated brain structure.

The ventral midbrain (including substantia nigra) was excluded from the ^1^H‐MRS analysis due to technical difficulties in reliably shimming in small, anatomically heterogeneous structures [38, 39]. Since acquisition was limited to areas that permitted stable and repeatable metabolite quantification in order to minimize anesthesia time and protect animal welfare. Additionally, acquisition from both striatal and ventral midbrain regions in a single session would have significantly extended scan duration.

T1‐weighted anatomical reference images obtained employing Rapid Imaging with Refocused Echoes (RARE) sequence in all three fundamental orientations (axial, coronal, and sagittal): To ensure exact voxel placement, the following parameters were used: field of view (FOV) = (37 × 37 mm^2^), image matrix = 256 × 256, slice thickness = 0.8 mm, repetition time (TR) = 1500 ms, and echo time (TE) = 5.8 ms. For the LdHIPP and RdHIPP, the voxel volumes were adjusted to 9 mm^3^ (1.5 mm × 1.5 mm × 4 mm), and for the treatment study, to 7.8 mm^3^ (1.5 mm × 1.3 mm × 4 mm). In both investigations, the investigated volume in the LsmCTX and RsmCTX was 12 mm^3^ (3 mm × 1 mm × 4 mm). Single‐voxel ^1^H‐MRS data were acquired using the point‐resolved spectroscopy (PRESS) sequence (Figure S2). This experiment included the subsequent parameters: spectrum width (SW) = 4401.41 Hz, 4096 points, TE = 16.5 ms, TR = 2500 ms, 256 averages; two 180° refocusing pulses had a Hermite shape and lasted 1.43 ms each, and a 90° excitation pulse had a length of 1 ms. The PRESS experiment included the following specifications: two 1.05‐ms‐long 180°‐calculated refocusing pulses, two 0.78‐ms‐long 90°‐calculated excitation pulses, 4096 points, TE = 16.5 ms, TR = 2500 ms, and 256 averages. The variable power and optimized relaxations delays (VAPOR)—outer volume suppression (OVS) and water suppression (WS) pulses—were used. Following each PRESS sequence, the quality of the spectra was validated visually during the ^1^H‐MRS data post‐processing and by measuring the linewidth of the water peak at 50% height [31].

#### 2.8.1 Data Processing

Using the spectra quality, all spectroscopic data were first sorted into categories of relevant and irrelevant data. The category data that was determined to be irrelevant was excluded from further post‐processing and statistical analysis. The average line width in 50% of the water peak height in the relevant category was 16.0 Hz, 2.0 Hz in the cortex voxels, and 12.3 Hz, 1.5 Hz in the HIPP voxels. The free clinical and biological ^1^H‐MRS program jMRUI was used to process these data [40]. Using the identical PRESS sequence, the basis set for the identified major brain metabolites creatinine (Cr), phosphorylated creatinine (PCr), N‐acetyl aspartate (NAA), choline (Cho), glutamate (Glu), glutamine (Gln), myoinositol (mI), and taurine (Tau) in NMRScopeB was simulated [41].

The basis sets were utilized in the model function of the completely automated QUEST‐MM: jMRUI quantitation method [42]. Before quantitation, the SVD algorithm in jMRUI eliminated all residual water from each spectrum. To compare the obtained spectra and determine the relative proportions of the metabolites, QUEST‐MM automatically determined the background (lipids and macromolecules) based on their physical model. Metabolite concentration ratios (Cho/NAA, Cho/tCr, NAA/tCr, Glx/tCr, mI/tCr, and Tau/tCr, where tCr = Cr + PCr and Glx = Glu + Gln) were calculated after acquiring the relative amplitudes of metabolites from each measured voxel. These ratios can serve as markers for neuronal loss (Cho/NAA), membrane turnover (Cho/tCr), neuronal integrity (NAA/tCr), astrogliosis (Glx/tCr), gliosis (mI/tCr), and osmoregulation (Tau/tCr). Hence, they were used to evaluate metabolite differences between the saline and the MPTP group.

### 2.9 Immunofluorescence Staining

Immunofluorescence staining and analyses were conducted on the brain sections to correlate with the markers from magnetic resonance imaging studies. Following MRI and ^1^H‐MRS acquisition, animals were anesthetized by administering xylazine (10 mg/kg, Rometar) and ketamine (100 mg/kg, Narketan) intraperitoneally. After anesthetizing, animals were transcardially perfused with saline and 4% paraformaldehyde in 0.1 M phosphate buffer (pH 7.4). The brain was isolated and immersed in 4% paraformaldehyde solution overnight at 4°C and then transferred into 15% sucrose solution for one day and then 30% sucrose solution for another day. After that, the brain was embedded in an OCT compound and stored at −80°C. The embedded brains were cryosectioned coronally at 10 μm thickness on coated slides using Cryostat CM3050S, Leica Microsystems. Slides with sections were stored at −20°C for further histological analysis.

A systematic set of 3 sections per region per animal was considered based on the MR imaging for the immunofluorescence analysis to correlate the findings. The sections were air dried for 15 min and post‐fixed with 4% paraformaldehyde for 15 min. Non‐specific binding sites were blocked with protein block (Ab647226; Abcam) for 1 h at room temperature. Sections were incubated overnight with primary antibodies [rabbit anti‐TH antibody (1:500), chicken NeuN antibody (1:500), rabbit anti‐GFAP antibody (1:1000), and rabbit anti‐IBA1 (1:2000) antibody]. After washing with PBS, sections were washed again with PBS and incubated with species‐specific secondary antibodies conjugated to fluorescent dyes [Alexa Fluor 555 donkey anti‐rabbit IgG (1:500) and Alexa Fluor 647 goat anti‐chicken IgY (1:500)] for 1 h at room temperature in the dark. The sections were incubated with DAPI for 20 min after secondary antibody incubation. At last, the sections were mounted in mowiol and the coverslipped. The fluorescence images were captured by the microscope slide scanner, Carl Zeiss. TH and NeuN were quantified by Image Pro‐Premier (IPP.v9.1.5), whereas GFAP and IBA1 were quantified by ImageJ software [26, 43].

### 2.10 Statistical Data Analysis

Primary data were summarized using the arithmetic mean and standard error of mean (±SEM). The Shapiro–Wilk test was used to test the data for normality, and data were analyzed by appropriate non‐parametric tests when normality was not confirmed. Behavioral analyses were calculated using IBM SPSS Statistics 28.0.0.0 (190) using a mixed model for repeated measures in OFT timeline data, and an unpaired *t*‐test for group comparisons. DKI data was analyzed. ^1^H‐MRS and immunofluorescence data were analyzed by unpaired two‐tailed Student's *t*‐test. GraphPad Prism 8.0 was used to perform the statistical analysis. A value of *p* < 0.05 was set as the boundary of statistical significance in all applied tests.

## 3 Results

### 3.1 MPTP Impaired the Motor Coordination and Neuromuscular Strength

First, motor impairment induced by intraperitoneal administration of MPTP was examined at the 72‐h time point by grid test, pole test, and OFT. A significant decrease in latency to fall was observed in the grid test in the MPTP group as compared to the saline group (*p* < 0.01). However, we observed no significant differences in motor abilities (pole test and OFT), nor memory impairment (Y‐maze test) (Figure 1).

### 3.2 DKI Detected the Microstructural Changes Induced by MPTP

To investigate the MPTP‐induced microstructural changes, diffusion and kurtosis metrics were analyzed by DKI. At the 24‐h time‐point, ROI analysis revealed significantly higher mean (*p* < 0.05), axial (*p* < 0.01), and radial (*p* < 0.05) diffusivity in HIPP and MD (*p* < 0.05) in THAL in the MPTP‐treated mice compared to saline‐treated mice (Table 1).

**TABLE 1:** DKI ROI analysis at 24‐h time‐point in different regions.

ROI	Group	Kurtosis Metrics				Diffusivity Metrics (in mm^2^/s)		
		Fractional Anisotropy (FA)	Mean Kurtosis (MK)	Axial Kurtosis (AK)	Radial Kurtosis (RK)	Mean Diffusivity (MD)	Axial Diffusivity (AD)	Radial Diffusivity (RD)
STR	Saline	0.18 ± 0.00	0.80 ± 0.01	0.91 ± 0.01	0.74 ± 0.02	0.00068 ± 0.00001	0.00080 ± 0.00001	0.00061 ± 0.00001
	MPTP	0.18 ± 0.01	0.79 ± 0.02	0.88 ± 0.02	0.76 ± 0.02	0.00070 ± 0.00001	0.00083 ± 0.00002	0.00063 ± 0.00001
HIPP	Saline	0.20 ± 0.00	0.77 ± 0.01	0.84 ± 0.01	0.74 ± 0.01	0.00077 ± 0.00002	0.00092 ± 0.00002	0.00069 ± 0.00002
	MPTP	0.20 ± 0.01	0.75 ± 0.02	0.81 ± 0.01	0.74 ± 0.04	0.00089 ± 0.00003*	0.00106 ± 0.0003**	0.00080 ± 0.00003*
smCTX	Saline	0.16 ± 0.00	0.69 ± 0.01	0.82 ± 0.01	0.63 ± 0.01	0.00070 ± 0.00002	0.00082 ± 0.00002	0.00064 ± 0.00001
	MPTP	0.16 ± 0.01	0.68 ± 0.01	0.80 ± 0.01	0.61 ± 0.01	0.00073 ± 0.00001	0.00084 ± 0.00001	0.00066 ± 0.00001
Thalamus	Saline	0.27 ± 0.00	0.83 ± 0.03	0.91 ± 0.02	0.78 ± 0.04	0.00071 ± 0.00001	0.00091 ± 0.00001	0.00061 ± 0.00001
	MPTP	0.27 ± 0.01	0.81 ± 0.01	0.88 ± 0.02	0.76 ± 0.03	0.00075 ± 0.00002*	0.00096 ± 0.00002*	0.00064 ± 0.00002
SN	Saline	0.31 ± 0.01	1.00 ± 0.03	0.99 ± 0.02	1.02 ± 0.05	0.00078 ± 0.00002	0.00104 ± 0.00003	0.00064 ± 0.00002
	MPTP	0.32 ± 0.01	0.98 ± 0.01	0.95 ± 0.01	1.00 ± 0.02	0.00083 ± 0.00003	0.00113 ± 0.00003	0.00068 ± 0.00003

*Note:* The table presents the analysis of DKI performed at 24‐h timepoint (number of subjects was saline: *n* = 7, MPTP: *n* = 9). The results are mean ± SEM, results of *t*‐test in asterisk (*p < 0.05; **p < 0.01; ***p < 0.001). Kurtosis and fractional anisotropy are dimensionless units; diffusivity values are given in mm^2^/s.

Abbreviations: MK, mean kurtosis; AK, axial kurtosis; RK, radial kurtosis; MD, mean diffusivity; AD, axial diffusivity; RD, radial diffusivity; FA, fractional anisotropy.

At the 72‐h time‐point, we observed significantly higher mean (*p* < 0.05), axial (*p* < 0.05), and radial (*p* < 0.05) diffusivity in STR, RD in THAL (*p* < 0.05), AD (*p* < 0.05) in HIPP, and smCTX (*p* < 0.05) of the MPTP‐treated mice compared to saline control (Table 2).

**TABLE 2:** DKI ROI analysis at 72‐h time‐point in different regions.

ROI	Group	Kurtosis metrics				Diffusivity metrics (mm^2^/s)		
		Fractional anisotropy (FA)	Mean kurtosis (MK)	Axial kurtosis (AK)	Radial kurtosis (RK)	Mean diffusivity (MD)	Axial diffusivity (AD)	Radial diffusivity (RD)
STR	Saline	0.17 ± 0.01	0.80 ± 0.01	0.90 ± 0.02	0.75 ± 0.01	0.00067 ± 0.00000	0.00079 ± 0.00001	0.00061 ± 0.00000
	MPTP	0.18 ± 0.00	0.79 ± 0.01	0.88 ± 0.01	0.77 ± 0.01	0.00070 ± 0.00001*	0.00083 ± 0.00001*	0.00063 ± 0.00001*
HIPP	Saline	0.19 ± 0.00	0.78 ± 0.01	0.84 ± 0.01	0.76 ± 0.01	0.00076 ± 0.00001	0.00091 ± 0.00001	0.00061 ± 0.00000
	MPTP	0.19 ± 0.00	0.77 ± 0.02	0.84 ± 0.01	0.77 ± 0.01	0.00079 ± 0.00001	0.00095 ± 0.00001*	0.00064 ± 0.00001
smCTX	Saline	0.16 ± 0.00	0.69 ± 0.00	0.81 ± 0.01	0.62 ± 0.01	0.00070 ± 0.00001	0.00081 ± 0.00001	0.00064 ± 0.00001
	MPTP	0.16 ± 0.00	0.65 ± 0.04	0.81 ± 0.01	0.63 ± 0.01	0.00072 ± 0.00001	0.00084 ± 0.00001*	0.00066 ± 0.00001
Thalamus	Saline	0.26 ± 0.00	0.85 ± 0.01	0.91 ± 0.01	0.81 ± 0.02	0.00070 ± 0.00001	0.00089 ± 0.00001	0.00060 ± 0.00000
	MPTP	0.26 ± 0.01	0.85 ± 0.02	0.92 ± 0.01	0.80 ± 0.01	0.00072 ± 0.00001	0.00091 ± 0.00001	0.00062 ± 0.00000*
SN	Saline	0.32 ± 0.01	1.03 ± 0.01	0.98 ± 0.01	1.06 ± 0.02	0.00081 ± 0.00001	0.00109 ± 0.00002	0.00067 ± 0.00001
	MPTP	0.31 ± 0.01	1.01 ± 0.01	0.99 ± 0.01	1.05 ± 0.02	0.00081 ± 0.00001	0.00109 ± 0.00002	0.00068 ± 0.00001

*Note:* The table presents the analysis of DKI performed at 72‐h timepoint (number of subjects was saline: *n* = 7, MPTP: *n* = 9). The results are mean ± SEM, results of *t*‐test in asterisk (*p < 0.05; **p < 0.01; ***p < 0.001). Kurtosis and fractional anisotropy are dimensionless units; diffusivity values are given in mm^2^/s.

Abbreviations: MK, mean kurtosis; AK, axial kurtosis; RK, radial kurtosis; MD, mean diffusivity; AD, axial diffusivity; RD, radial diffusivity; FA, fractional anisotropy.

### 3.3 ^1^H‐MRS Revealed MPTP‐Induced Metabolic Alterations in the Striatum and Hippocampus

**FIGURE 2:**
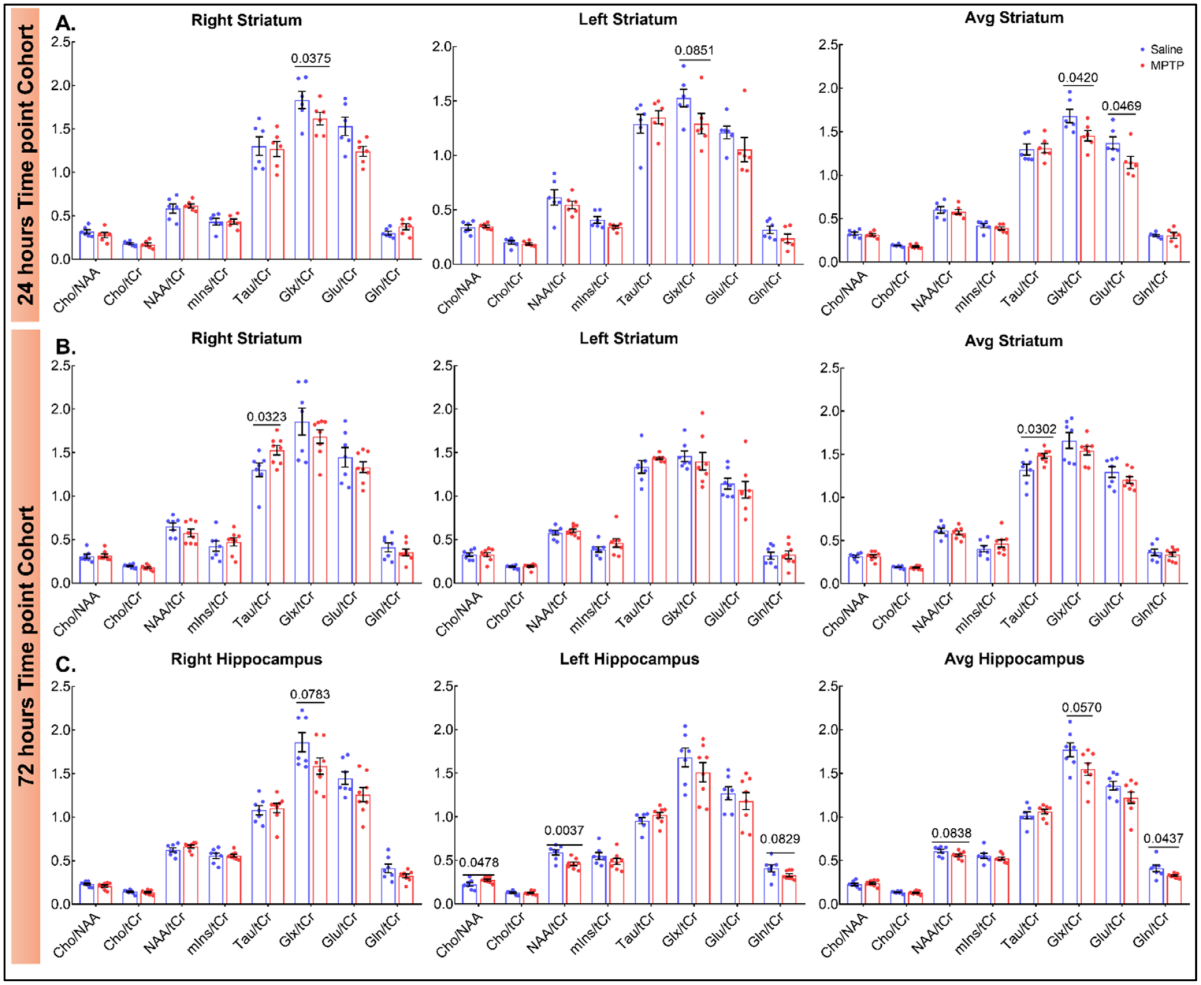
^1^H MRS detected the MPTP‐induced metabolite alterations. (A) Bar graphs represent metabolite ratios in the striatum in 24 h cohort. (B, C) Bar graphs represent the metabolite ratios in the striatum and hippocampus in 72 h cohort, respectively. Data are expressed as mean values ± SEM, *n* = 6 for 24 h and *n* = 7–8 for 72‐h time‐point, respectively.

Next, to detect neurochemical changes associated with MPTP administration, ^1^H‐MRS was performed on the STR and HIPP. We observed significantly lower Glu/tCr (*p* < 0.05) and Glx/tCr ratios (*p* < 0.05) in the STR of the MPTP‐treated mice at the 24‐h time point (Figure 2A).

At the 72‐h time‐point, ^1^H‐MRS revealed a significant elevation in average taurine (Tau/tCr; *p* < 0.05) in STR (Figure 2B). ^1^H‐MRS in the HIPP documented significantly higher Gln/tCr (*p* < 0.05) and Cho/NAA (*p* < 0.05) ratios, while NAA/tCr (p < 0.05) was found lower in the left HIPP in MPTP‐treated mice, suggesting lateralization of the pathological process (Figure 2C).

### 3.4 MPTP‐Induced Neuronal Loss and Astrocyte Activation in Striatum and Hippocampus

**FIGURE 3:**
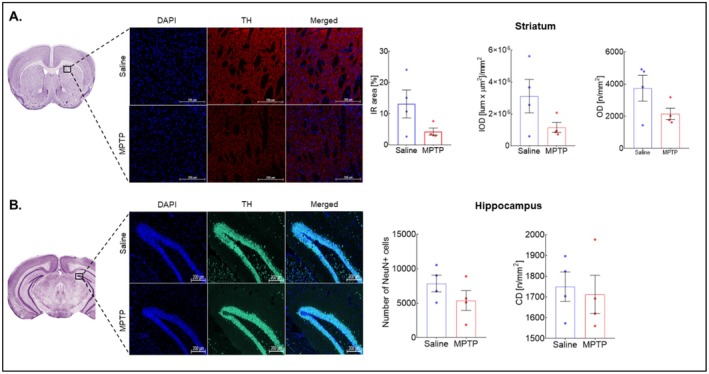
Effect of MPTP on TH and NeuN expression after 24 h. Representative images and graphs of the TH expression in (A) striatum and (B) NeuN expression in hippocampus, respectively. Data were reported as mean ± SEM (*n* = 4–5). Abbreviations: IR Immunoreactivity; IOD, Integrated Optical Density; OD Object Density; CD Cell Density.

Next, in order to correlate the DKI findings, we assessed the neurodegeneration and glial activation by immunofluorescence. At the 24‐h time‐point, the number of NeuN‐positive cells in HIPP and TH expression in STR were found to be lower in the treated group, but not significantly compared to the saline group (Figure 3a,B).

**FIGURE 4:**
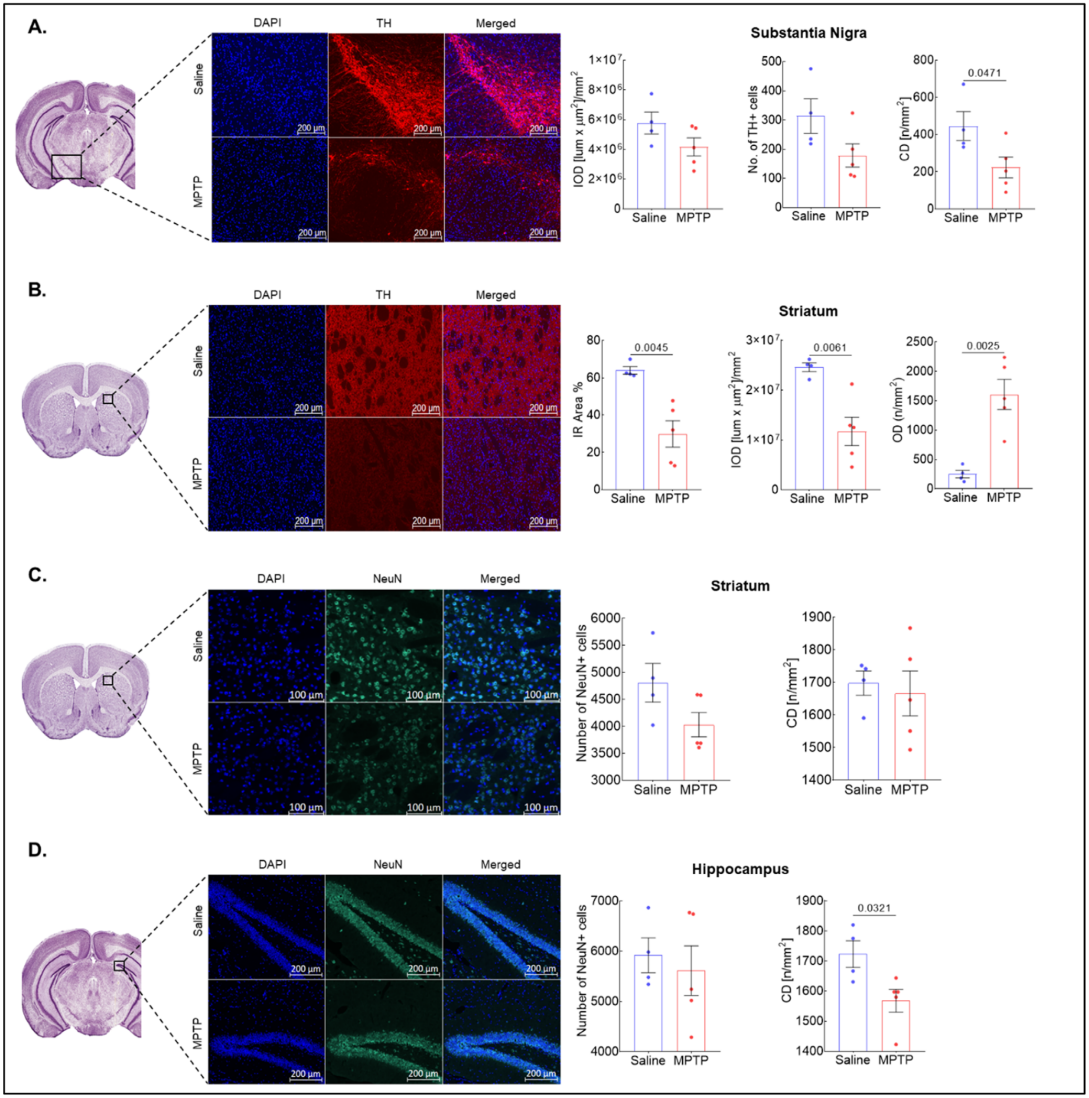
Effect of MPTP on TH and NeuN expression after 72 h. (A, B) Representative images and graphs of the TH expression in substantia nigra and striatum, respectively. (C, D) Representative images and graphs of the NeuN expression in striatum and hippocampus, respectively. Data were reported as mean ± SEM (*n* = 4–5). Abbreviations: IR Immunoreactivity; IOD, Integrated Optical Density; OD Object Density; CD Cell Density.

**FIGURE 5:**
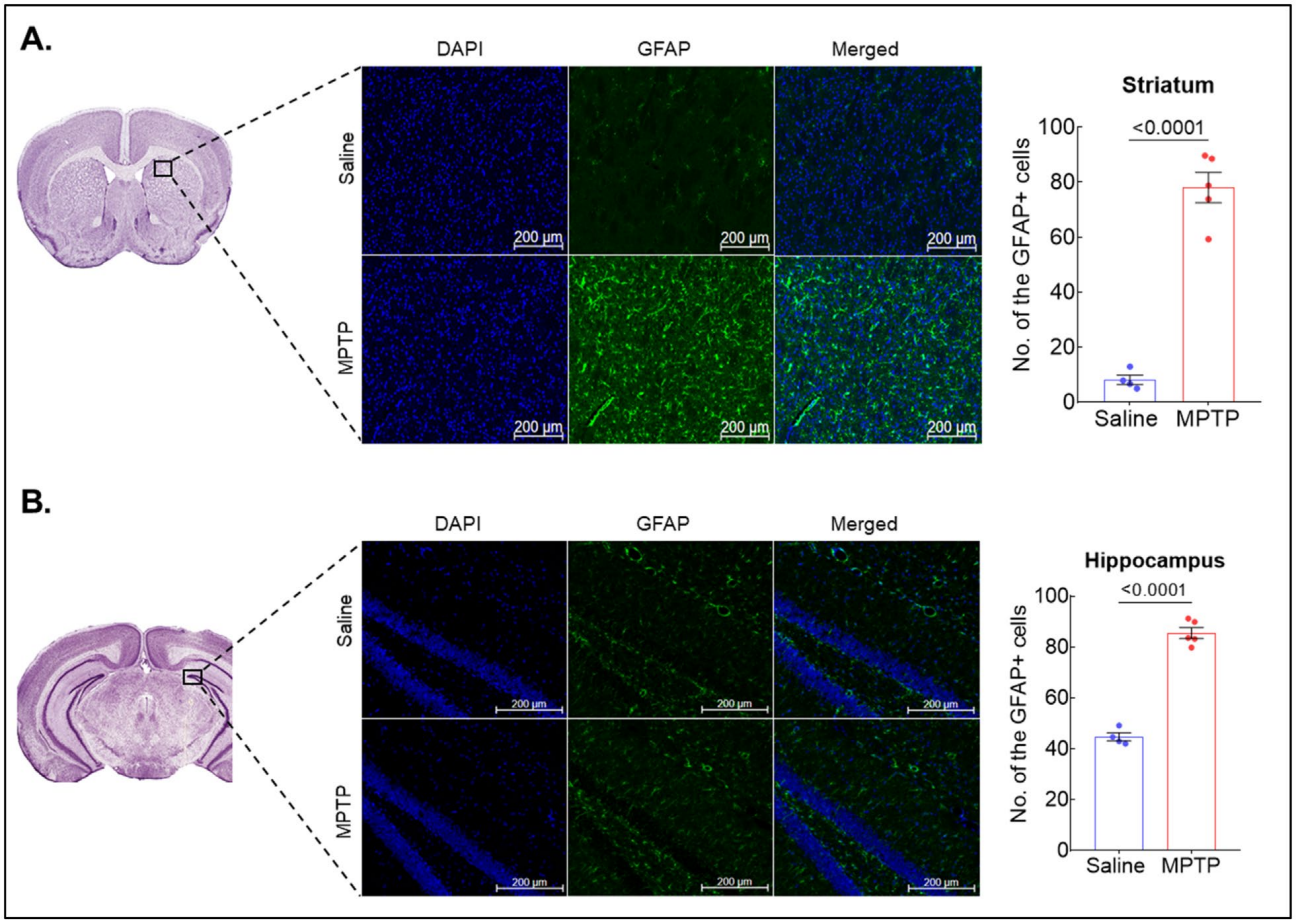
Effect of MPTP on GFAP expression after 72 h. Representative images of immunofluorescence staining for GFAP expression and graphs representing GFAP+ cells in (A) striatum and (B) hippocampus, respectively. Data were reported as mean ± SEM (*n* = 4–5).

At the 72‐h time‐point, a significant decrease in TH expression was observed in the STR (*p* < 0.05) of the MPTP group (Figure 4B). There was also a decrease in the expression of TH in SN of this group; however, it was not significant, though cell density was found to be significantly decreased (Figure 4A). Additionally, NeuN staining in HIPP revealed a significant decrease in the cell density in the MPTP group (*p* < 0.05) as compared to the saline group (Figure 4D). A decrease in cell density with NeuN staining was also noted in STR of the MPTP group as compared to saline, though it was not significant (Figure 4C). Interestingly, a highly significant difference was observed in the expression of GFAP in both STR (*p* < 0.001) and HIPP (p < 0.001), suggesting the activation of astrocytes in the MPTP group (Figure 5A,B). No effect was observed in THAL and smCTX. However, there was no change in IBA1 expression among both groups (Figure S3).

### 3.5 TBSS Revealed MPTP‐Induced Diffusivity Changes in Mice Brain

**FIGURE 6:**
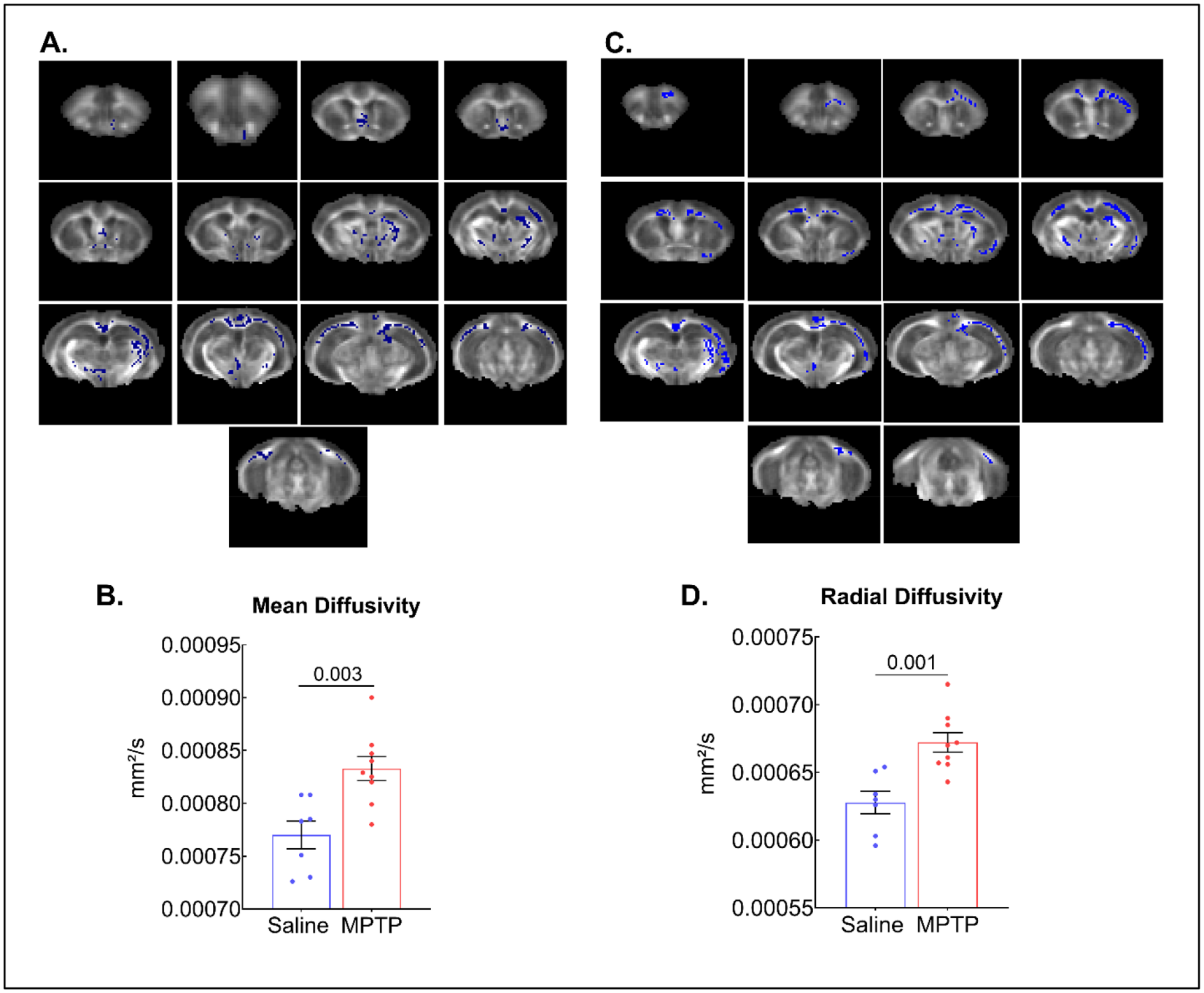
TBSS analysis in mice after 72 h of MPTP treatment. (A, C) Schematic representation of TBSS analysis for mean and radial diffusivity, respectively. (B, D) TBSS analysis showed significantly higher values in mean and radial diffusivity extracted from all significant areas in the TBSS analysis (i.e., the regions highlighted in blue in panels A and C), in the cingulate gyrus, subgeniculate nucleus of prethalammus, ventral tegmental area of the rostral part, molecular layer of the dentate gyrus, ventral posterior nucleus of the thalamus, internal capsule, fimbria of the hippocampus, and medial septal nucleus. Data were reported as mean ± SEM (*n* = 7–9).

Finally, whole‐brain TBSS MRI analysis was conducted to identify widespread microstructural alterations. TBSS MRI performed at 72‐h time‐point showed significantly higher MD (*p* < 0.01) and RD (*p* < 0.01) in the MPTP‐treated mice (Figure 6). These findings correspond to diffusivity changes observed in the Cingulate gyrus, Subgeniculate nucleus of the prethalammus, ventral tegmental area of the rostral part, molecular layer of the dentate gyrus, ventral posterior nucleus of the THAL, internal capsule, fimbria of the HIPP, and medial septal nucleus of the MPTP group.

## 4 Discussion

In the present study, we examined the microstructural and metabolic changes in mice after repeated systemic administration of MPTP. MPTP exhibits a selective neurotoxic effect on the dopaminergic neurons located in the SN, culminating in neurodegenerative processes and the hallmark symptoms associated with PD [44, 45]. Consistent with the literature, repeated MPTP doses in this study induced a significant reduction in dopaminergic neuronal terminals in the striatum, which were concurrently linked to impaired motor performance and changes in behavioral activity [46].

A significant reduction in latency to fall was observed in the grid test among the MPTP‐treated cohort, indicating the emergence of motor function impairments associated with PD in this group. The motor function deficit detected in the grid test can be corroborated by the reduced TH expression observed with immunohistochemistry in SN and STR of the MPTP‐treated mice in our study. However, no notable differences were detected in other behavioral assessments, which can be attributed to the high sensitivity of the grid test in detecting motor impairments during the initial stages of PD onset [14, 15, 26, 47].

We observed a significant increase in MD in HIPP and THAL in the 24‐h time‐point. These findings can be backed by a study wherein acute MPTP treatment led to a significant decrease in spine density of neuronal dendrites in HIPP of these mice, which supports increased diffusivity observed in the MPTP‐treated group in our study [45]. This acute increase in diffusivity can be associated with the higher sensitivity of HIPP and THAL to the neurotoxin‐mediated insult, leading to neurodegeneration. A study performed on C57BL/6 mice using i.p. injection of MPTP for 5 consecutive days reported significant upregulation of phosphorylated α‐syn, α‐syn, p‐Tau, and Tau proteins, showcasing the sensitivity of HIPP to MPTP‐induced neurotoxicity [48]. In another study, a single 50 mg/kg dose of i.p. injected MPTP precipitated neurodegeneration in THAL of CD‐1 mice, supporting early diffusivity differences observed at the 24‐h timepoint [49]. We also observed a decrease in neuronal expression detected through NeuN staining, though not significant in HIPP at 24 h, further supporting the diffusivity differences observed at this time point.

However, at the 72‐h time point, there was a significant difference in only AD in HIPP and RD in THAL of MPTP‐treated mice, compared to the 24‐h time point, where all the diffusivity metrics, MD, AD, and RD, were significantly higher. This apparent recovery in diffusivity, particularly in the hippocampus, may reflect a region‐specific compensatory response. Previous studies have demonstrated that the hippocampus is particularly susceptible to early MPTP‐induced toxicity, yet it also exhibits a degree of plasticity and regenerative potential, including dendritic remodeling and spontaneous sprouting [45, 48]. Peng and Andersen (2011) reported that acute MPTP exposure in young adult wild‐type mice significantly increased BrdU/DCX double‐positive cells in the subventricular zone of the HIPP, indicating enhanced proliferation of nascent neurons in response to injury. While their study focused on the subventricular zone, it reinforces the notion that acute MPTP insult can stimulate endogenous regenerative responses in neurogenic regions of the brain [50]. Furthermore, we used adult male mice whose regenerative potential is higher than in elderly mice. Age and sex have been shown to influence brain function and cognitive performance in mice [51]. In our study, this may be reflected by the normalization of hippocampal diffusivity and concurrent upregulation of GFAP expression, possibly signifying an early gliotic and neurogenic response. In contrast, such regenerative potential is limited in the substantia nigra and striatum, where we observed ongoing neurodegeneration at 72 h, evidenced by decreased dopaminergic and neuronal expression and supported by prior findings of dopaminergic terminal loss and gliosis in these areas [52, 53, 54]. Therefore, the recovery pattern observed in the hippocampus likely reflects region‐specific neuroplasticity that is not paralleled in nigrostriatal areas, which continue to degenerate. Moreover, this regenerative potential of the neuronal system can be attributed to numerous factors, such as GM1 ganglioside [53, 55], cerebral DA neurotrophic factor [56], or mesencephalic astrocyte‐derived neurotrophic factor [57].

We observed an increase in all diffusivity metrics in STR of MPTP‐treated mice at 72‐h time‐point. Similar findings have been reported in early diagnosis of PD patients wherein DKI revealed significantly higher diffusivity in putamen and caudate compared to healthy age‐matched controls [58]. Numerous studies have reported that striatal DA denervation appears to be related to the reduced dendritic length and spine number in this brain region in both patients with PD and MPTP‐lesioned animal models [54, 59, 60]. The increased diffusivity in STR observed with DKI is in line with our immunohistochemistry results, where we observed a significant decrease in TH immunoreactivity in the striatum compared to saline‐treated mice. Surprisingly, though we observed a decrease in TH immunoreactivity in SN, we were not able to observe any significant diffusivity changes with our DKI imaging.

TBSS analysis, which detects white matter changes, was unable to show any kurtosis and diffusivity changes at the earlier time point (24 h). However, at the 72‐h time point, we found an increase in MD and RD in white matter regions such as the dentate gyrus, cingulate gyrus, internal capsule, and ventral tegmental area. Similar to our results, patients suffering from Parkinson's disease with dementia also showed increased MD in the internal capsule [61]. Several studies detecting white matter integrity in PD patients have suggested that white matter changes often precede gray matter changes [62]. Unfortunately, the present DKI study was not able to detect the earlier white matter changes, but at the 72‐h time point, when gray matter changes were also visible.

With ^1^H‐MRS analysis, at a 24‐h time‐point, we observed a significantly decreased Glx/tCr ratio in the STR of MPTP‐treated mice. Similar to our findings, a study in rats lesioned by the injection of 6‐hydroxydopamine (6‐OHDA) in the medial‐forebrain bundle also reported significant decreases in Glu levels in the STR of experimental rodents [63]. Contrary to our results, studies have reported increased levels of Gln/tCr supporting gliosis and elevated Glu/tCr levels indicating changes in corticostriatal activity leading to increased synthesis and release of Glu in the synaptic terminal of the STR [63, 64]. In addition to this, another study published on rodents did not report any significant changes in Glu levels in 6‐OHDA lesioned rats [65]. Therefore, in our present study, drawing any inferences by observing the imbalance of a single metabolite at 24‐h timepoint is not feasible.

At 72‐h time‐point, we observed increased Tau/tCr ratio in STR of MPTP‐treated mice. Literature suggests the astrocytes primarily synthesize the taurine in the brain [66, 67]. This heightened taurine can be correlated to the activation of astrocytes in a defensive response to toxicity induced by MPTP [68] and osmolarity dysregulation in this ROI [69, 70]. Similarly significant increment in taurine was found in STR of 6‐OHDA lesioned [69, 70] and MPTP brains of rodents. We also observed decreased Gln/tCr ratio in HIPP at 72‐h time‐point. This could be linked to the imbalance in steady‐state glutamine‐glutamate‐GABA equilibrium. Glial Gln is the primary precursor for neuronal Glu and GABA, and the Gln‐Glu cycle is a critical pathway for neurotransmitter shuttling between astrocytes and neurons. We did not observe any significant decrease in Glu/tCr and Glx/tCr ratios, while the GABA/tCr ratio (inhibitory neurotransmission) was not measured. Therefore, drawing any definite inferences is not possible presently [69, 70]. A unilateral increase in Cho/tCr and NAA/tCr was also noted in HIPP. An increase in ratios of these two metabolites is correlated with membrane breakdown and can be supported by the significant decrease in cell density observed in HIPP by NeuN staining [18, 19]. Similarly, few studies have reported unilateral differences in metabolite ratios in other rodent disease models. Research on interhemispheric differences is usually neglected in preclinical studies and requires further attention, as disease‐related changes may be visible in both or either of them [71].

One of the major limitations of this study is that we could not observe any significant changes in SN, the region of the midbrain primarily associated with PD, with DKI at both time points. However, we were able to detect diffusivity changes in STR and HIPP. The other limitation is that we did not perform ^1^H‐MRS in HIPP at the 24‐h time‐point, making it difficult to correlate the findings of immunohistochemistry and DKI with this time‐point and to understand if there existed any differences in metabolite concentration in HIPP from the 72‐h time‐point. We also did not perform any non‐motor symptom‐related behavior study; hence, we cannot correlate the findings of HIPP with non‐motor impairment associated with PD.

## 5 Conclusion

In this study, we hypothesized that utilizing diffusion kurtosis imaging (DKI) and proton magnetic resonance spectroscopy (^1^H‐MRS) would enable the detection of MPTP‐induced neuroinflammatory, neurodegenerative, and metabolic changes in the brains of C57/BL6 mice following repeated administrations of MPTP treatment.

MPTP‐treated C57/BL6 mice exhibited motor impairment at 72 h. Neuroinflammation and neurodegeneration were observed exclusively at the 72‐h time point in the striatum and hippocampus, but not at the 24‐h time point in MPTP‐treated mice. DKI was able to detect early microstructural changes at the 24‐h time point in the hippocampus and thalamus by showing an increase in diffusivity, but not in the striatum, indicating regional sensitivity to MPTP‐induced neurotoxicity. DKI imaging showed increased diffusivity in the striatum of MPTP‐treated mice at 72 h corresponding with a decrease in TH immunoreactivity. ^1^H‐MRS detected reductions in metabolite ratios like NAA/tCr, signaling neuronal death in the hippocampus and striatum. We further postulated that integrating these advanced imaging techniques with behavioral assessments and histological validation would provide a comprehensive approach to reveal the progression of PD‐related pathology. These findings not only enhance our comprehension of PD pathophysiology but also lay the groundwork for the development of improved diagnostic strategies and therapeutic interventions in clinical settings.

## Funding

This work was written at Masaryk University as part of the project “Specific pharmacological research in pharmacokinetics, behavioural neuropsychopharmacology and personalised pharmacotherapy in oncology” number MUNI/A/1819/2025 with the support of the Specific University Research Grant, as provided by the Ministry of Education, Youth and Sports of the Czech Republic (MEYS CR) in the year 2026. NMR experiments were carried out at the ISI MR facility of the Czech‐BioImaging infrastructure, supported by grant LM2023050 of the MEYS CR. Amit Khairnar gratefully acknowledges support from project no. LX22NPO5107 (MEYS CR), financed by European Union−Next Generation EU. TFO is supported by DFG SFB1286, Project B8. This work was also supported by Specific University Research Grant, as provided by the Ministry of Education, Youth and Sports of the Czech Republic in the year 2026 (MUNI/A/1795/2025).

## Ethics Statement

All animal experiments were conducted in accordance with EU Directive 2010/63/EU and the Czech Animal Protection Act No. 246/1992. The study was approved by the Animal Care Committee of the Faculty of Medicine, Masaryk University, the Animal Care Committee of the Czech Academy of Sciences, and the Czech Governmental Animal Care Committee. The work is reported in compliance with the ARRIVE guidelines.

## Conflicts of Interest

The authors declare no conflicts of interest.

## Supporting information


**Figure S1:** Illustration of region of interest delineation of the substantia nigra, hippocampus, thalamus, striatum, and sensory motor cortex according to the Paxinos Mouse Brain Atlas overlaid on fractional anisotropy maps (slice thickness: 500 μm). Color codes: substantia nigra, orange; hippocampus, yellow; thalamus, green; striatum, red; and sensorimotor cortex, blue.
**Figure S2:** Representative images of 1H‐ MRS spectra of striatum and hippocampus: (A) Top—reference anatomical images and the position of the MRS voxel in the left striatum. Bottom—representative NMR spectrum of the striatum. (B) Top—reference anatomical images and the position of the MRS voxel in the left hippocampus. Bottom—representative NMR spectrum of the striatum.
**Figure S3:** Effect of MPTP on IBA1+ cells in striatum and hippocampus of mice after 72 h of last dose administration. (A‐B) Representative images of immunofluorescence staining for IBA1 expression in striatum and hippocampus, of mice respectively. Bar graphs represent the number of IBA1+ cells in striatum and hippocampus in both groups. Data were reported as mean ± SEM (*n* = 3).

## Data Availability

The data that support the findings of this study are available from the corresponding author upon reasonable request.
