# Supplementary material for: Brain Alterations Linked to the MPTP Mouse Model of Parkinson's Disease Uncovered by Diffusion Kurtosis Imaging and Magnetic Resonance Spectroscopy
Source: CNS Neurosci Ther. 2026 Apr 20;32(4):e70846. doi: 10.1002/cns.70846 (PMC13093730; doi:10.1002/cns.70846)
Supplement: Supplementary file 1 — Figure S1: Illustration of region of interest delineation of the substantia nigra, hippocampus, thalamus, striatum, and sensory motor cortex according to the Paxinos Mouse Brain Atlas overlaid on fractional anisotropy maps (slice thickness: 500 μm). Color codes: substantia nigra, orange; hippocampus, yellow; thalamus, green; striatum, red; and sensorimotor cortex, blue. Figure S2: Representative images of 1H‐ MRS spectra of striatum and hippocampus: (A) Top—reference anatomical images and the position of the MRS voxel in the left striatum. Bottom—representative NMR spectrum of the striatum. (B) Top—reference anatomical images and the position of the MRS voxel in the left hippocampus. Bottom—representative NMR spectrum of the striatum. Figure S3: Effect of MPTP on IBA1+ cells in striatum and hippocampus of mice after 72 h of last dose administration. (A‐B) Representative images of immunofluorescence staining for IBA1 expression in striatum and hippocampus, of mice respectively. Bar graphs represent the number of IBA1+ cells in striatum and hippocampus in both groups. Data were reported as mean ± SEM (n = 3). [file CNS-32-e70846-s001.docx]

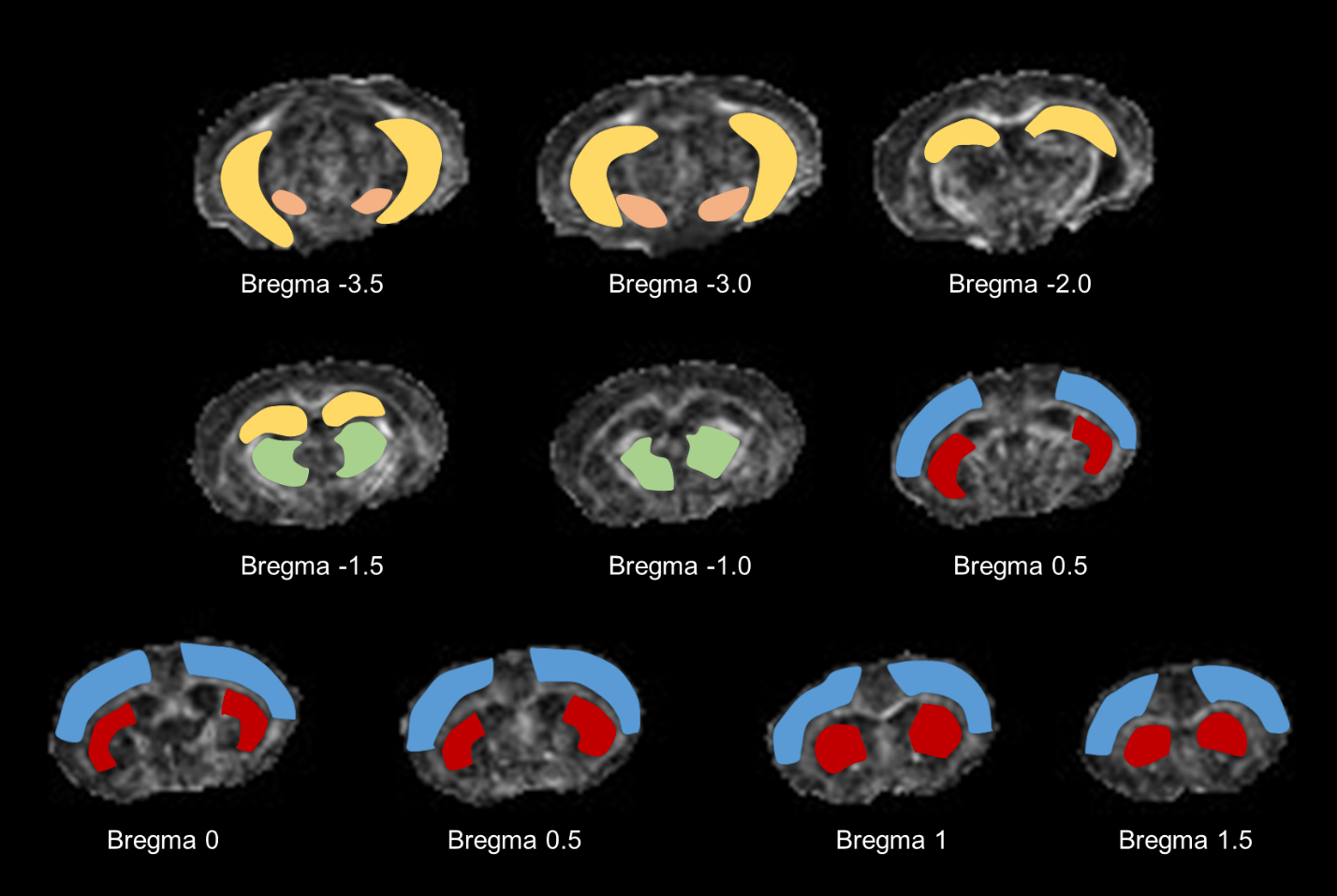


**Figure S1:** Illustration of region of interest delineation of the substantia nigra, hippocampus, thalamus, striatum, and sensory motor cortex according to the Paxinos Mouse Brain Atlas overlaid on fractional anisotropy maps (slice thickness: 500µm). Color codes: substantia nigra, orange; hippocampus, yellow; thalamus, green; striatum, red; and sensorimotor cortex, blue.


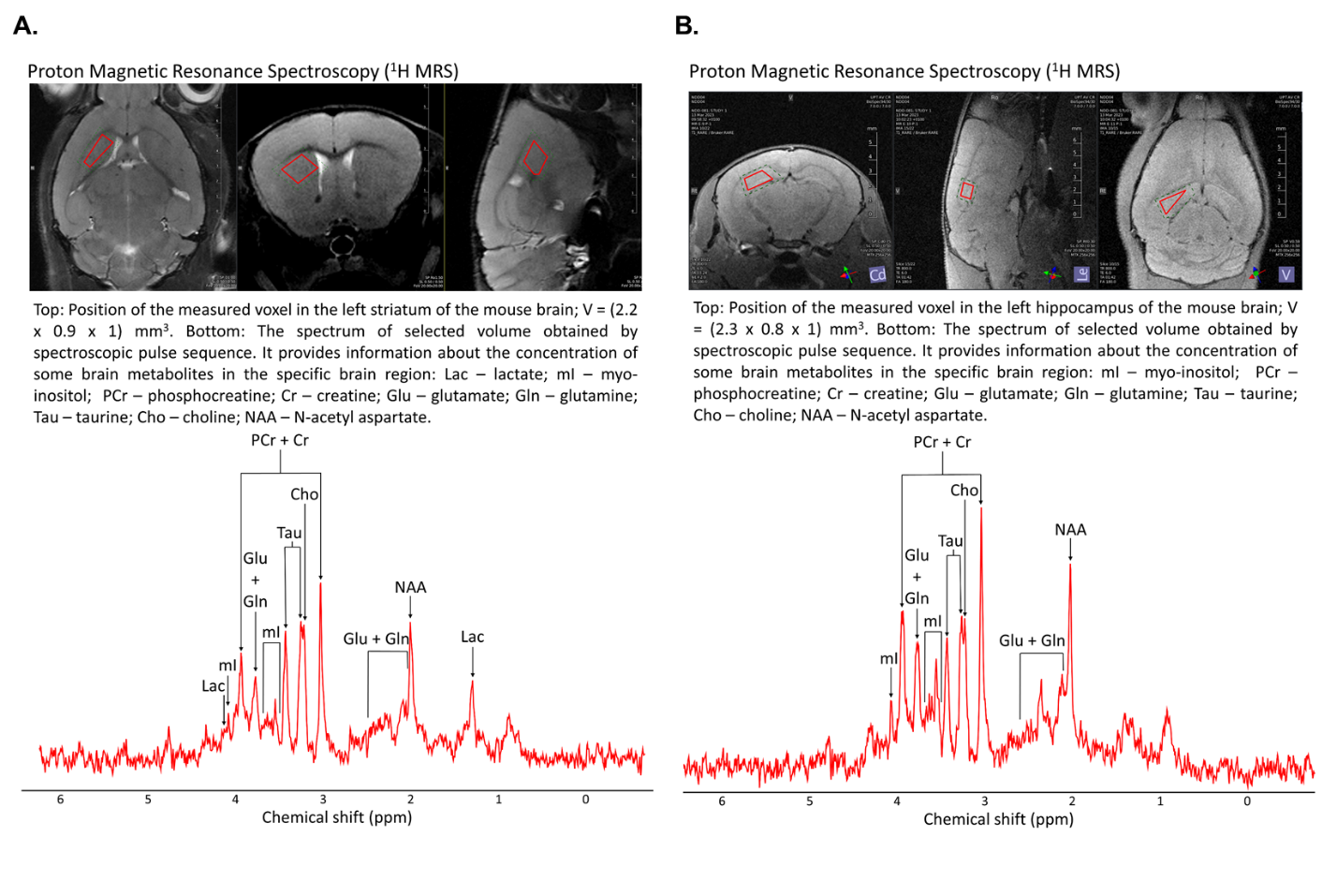


**Figure S2: Representative images of ^1^H- MRS spectra of striatum and hippocampus:** **(A)** Top – reference anatomical images and the position of the MRS voxel in the left striatum. Bottom - representative NMR spectrum of the striatum. **(B)** Top – reference anatomical images and the position of the MRS voxel in the left hippocampus. Bottom - representative NMR spectrum of the striatum.


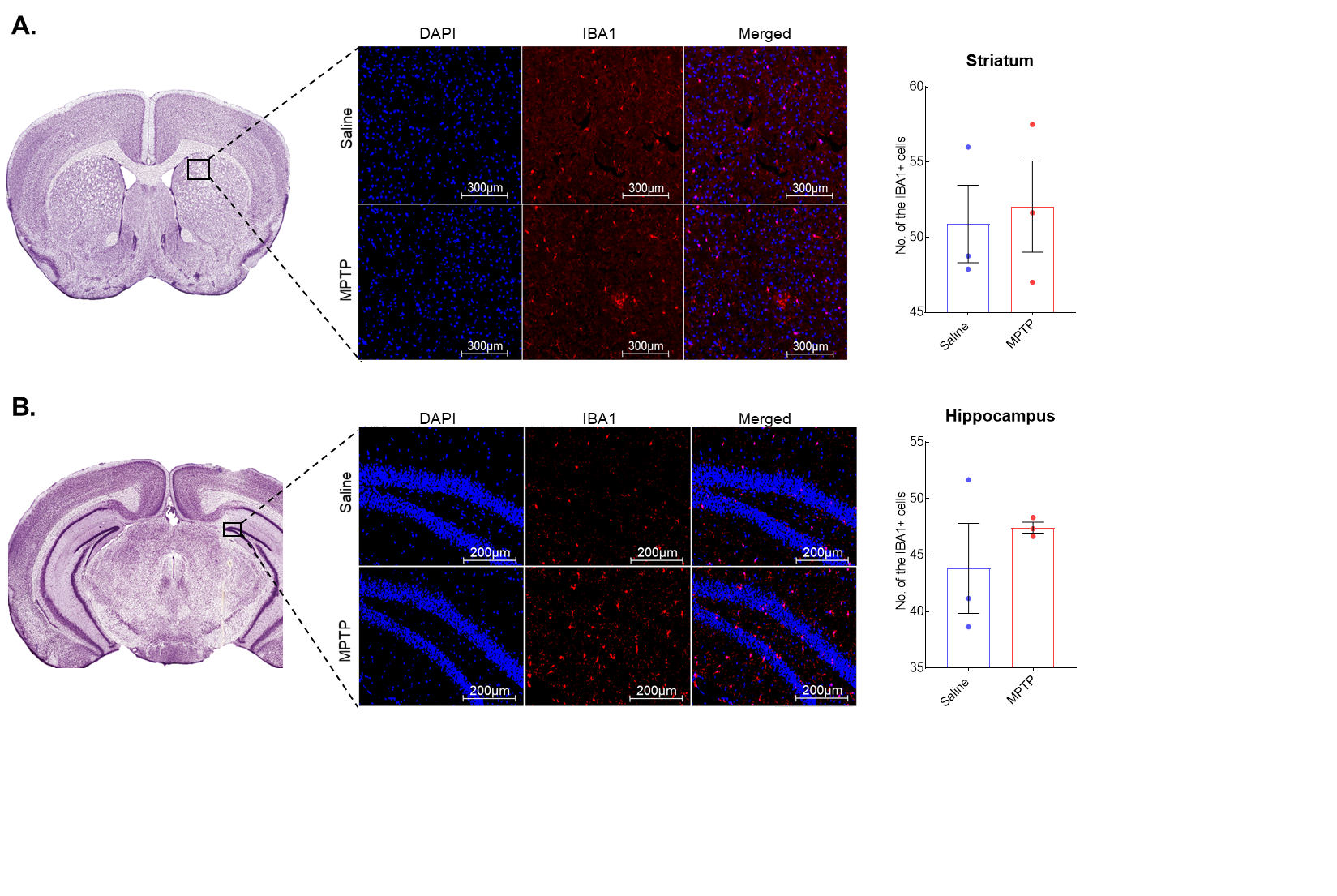


**Figure S3: Effect of MPTP on IBA1+ cells in striatum and hippocampus of mice after 72 hours of last dose administration.** (A-B) Representative images of immunofluorescence staining for IBA1 expression in striatum and hippocampus, of mice respectively. Bar graphs represent the number of IBA1+ cells in striatum and hippocampus in both groups. Data were reported as mean ± SEM (n=3).
